# Case Report: The Use of Rituximab in Antibody-Negative Autoimmune Encephalitis

**DOI:** 10.3389/fneur.2021.686009

**Published:** 2021-09-01

**Authors:** Soo-Hyun Park, Yong-Chan Kim

**Affiliations:** ^1^Department of Neurology, Department of Critical Care Medicine, Department of Internal Hospital, Inha University, Incheon, South Korea; ^2^Department of Neurology, Inha University Hospital, Incheon, South Korea

**Keywords:** autoimmune encephalitis, antibody-negative, brain MRI, CD19+/CD20+, rituximab, treatment

## Abstract

Antibody-negative autoimmune encephalitis (AE) is challenging to diagnose because clinically suspected antibody-negative AE cases are difficult to confirm. If not treated properly, like antibody-positive AE, antibody-negative AE can cause irreparable damage to patients. Previously, immunotherapy was effective in treating patients with antibody-negative AE. We present the case of a 63-year-old man who was admitted to our hospital with altered cognition. He was diagnosed with antibody-negative AE based on CSF, brain MRI, and B-cell counts; autoimmune diseases with similar clinical symptoms were ruled out. He was treated with immunotherapy, especially rituximab, for antibody-negative AE. After 3 weeks of treatment, his mental state and brain MRI results, concomitant with a decrease in CD19+/CD20+ B-cell counts. This case report shows that patients with antibody-negative AE may respond to rituximab, similar to those with antibody-positive AE. Thus, potentially undetected antibodies could be responsible for the treatment outcome.

## Introduction

Autoimmune encephalitis (AE) has recently emerged as a major cause of non-infectious encephalitis (1, 2). Because it is difficult to diagnose AE with only the clinical presentations, it is challenging to discern whether the symptoms are due to the underlying disease or triggered by auto-antibodies. Although AE diagnostic methods are advanced, the knowledge on antibody-based diagnosis is limited (1, 3). Moreover, cases of clinically suspected antibody-negative AE are difficult to confirm. Untreated, AE can cause irreversible neurological deficits. The management of antibody-negative AE is still not substantiated (1). However, immunotherapy was successful in 50% of antibody-negative AE patients suggesting that potentially undetected antibodies could be responsible for the treatment outcome (4). The repression of autoantibody production by long-lived plasma cells (half-life of > 6 months) was a key component of treatment. The efficacy of rituximab, an anti-CD20 B cell-targeting monoclonal antibody, was demonstrated by improving neurological symptoms and brain MRI findings (5). Mechanistically, rituximab lowered the systemic humoral immune response (6). Herein, we present a rare case of antibody-negative AE treated with rituximab, showing a correlation between the improved brain MRI results and decreased CD19+/CD20+ B-cell counts.

## Case Report

A 63-year-old man was admitted to our hospital with altered cognition for the previous 4 months. He was disoriented in time and space and unable to recall any words after 3 min; in addition, he had a progressive headache with nausea and vomiting for 1 week. He had no history of underlying diseases. The day after admission, he entered a state of stupor [Glasgow Coma Scale (GCS): E2V2M2] with respiratory failure.

**Figure 1:**
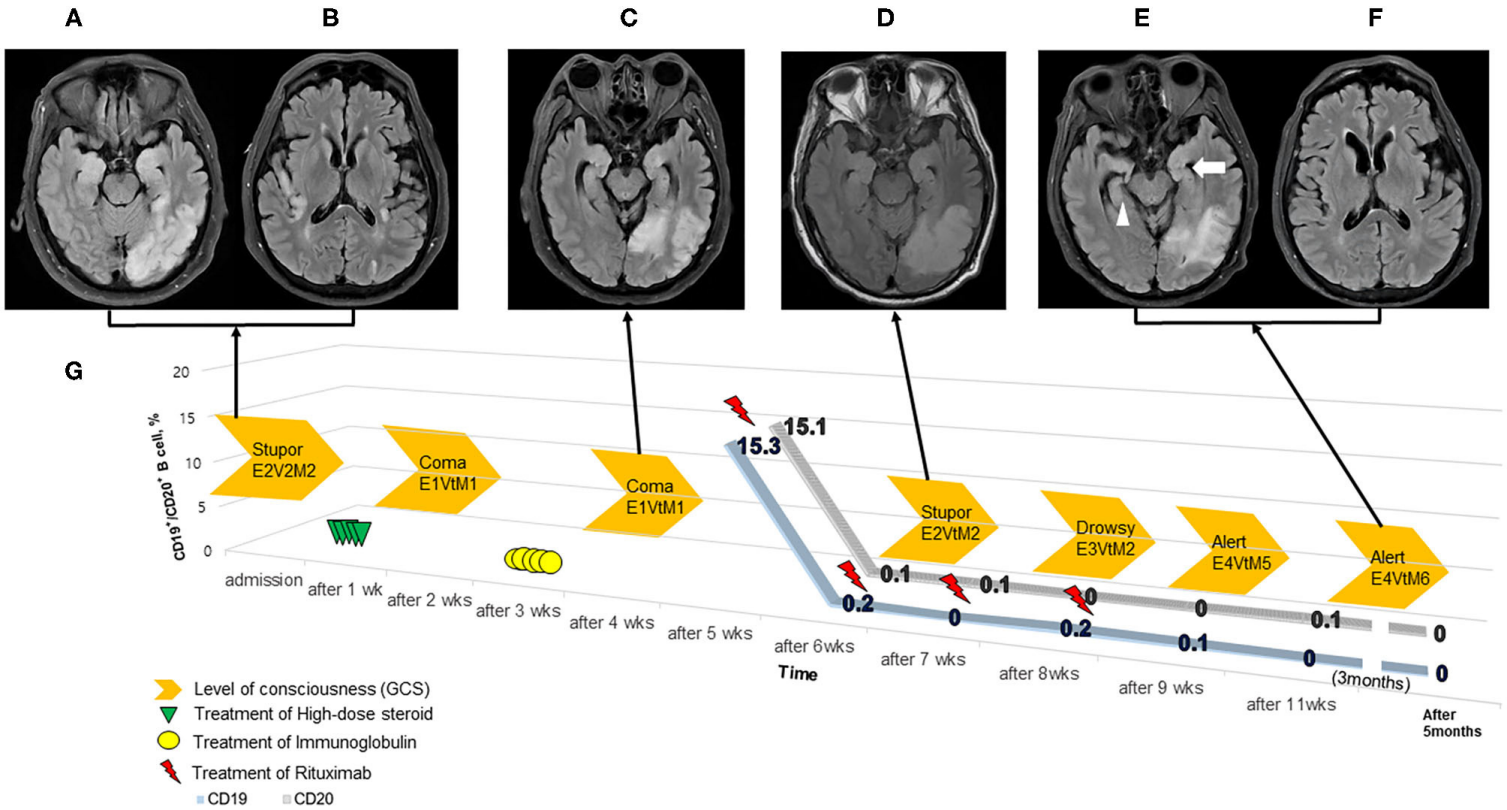
Timeline of the patient treatment. **(A–F)** Brain MRI (T2-FLAIR) demonstrates autoimmune encephalitis. The patient's level of consciousness (orange arrows, GCS) improved following treatment with high dose steroid (green triangle), intravenous immunoglobulin (yellow circle), and rituximab (red thunderbolt) **(G)**. The CD19^+^/CD20^+^ B-cell counts were present during the 5-week to 5-month follow-up after admission. There was the depletion of CD19^+^/CD20^+^ B-cell counts within 1 week after an initial infusion of rituximab.

Laboratory tests, including malignancy workup [i.e., Ri, Yo, Hu, Ma2/Ta (PNMA2), LGI1, CV2/CRMP5, amphiphysin, NMDAR, GABAR, recoverin, CASPR2, and AMPAR2] and vasculitis workup (i.e., ESR, CRP, RF, ANA, C3, C4, CH 50, ANCA, RPR/VDRL, protein electrophoresis) yielded negative results. Brain MRI (T2-FLAIR) revealed abnormal hyperintensity with expansion in the hippocampi, temporal cortex, medial thalami, right insular cortex, left pulvinar, and left parietal lobe, without restricted diffusion (not shown) or contrast enhancement (Figures 1A,B). Brain MRI (T2-FLAIR) demonstrates the disappearance of hyperintense and expansion lesions after treatment for autoimmune encephalitis, especially in the medial thalami, right insular cortex, and left pulvinar. Brain MRI (T2-FLAIR) also shows T2-FLAIR hyperintensity with atrophy in the left medial temporal lobe (white arrow), right medial temporal lobe (white arrowhead), and left parietal lobe (Figures 1C–F).

A lumbar puncture showed no evidence of infection (white blood cells: 0 cells/μL) or malignant cells. Blood culture, urine culture, and CSF were negative for HSV-1, HSV-2, HHV-3, HHV-6, HHV-7, HHV-8, Epstein-Barr virus, cytomegalovirus, Enterovirus, HBV, HIV, HTLV, *Treponema pallidum*, human polyomavirus 2, *Mycobacterium tuberculosis, Borrelia burgdorferi, Anaplasma phagocytophilum*, tick-borne encephalitis virus, and polyomavirus BK. The CSF was also negative for antibodies, including Ri, Yo, Hu, Ma2/Ta (PNMA2), LGI1, CASPR2, recoverin, Sox1, Titin, Zic4, DNER/Tr, amphiphysin, CV2/CRMP5, GAD65, NMDAR, GABAR, IgLON5, AMPAR2, DPPX, glycine receptor, and mGluR5. Finally, additional auto-antibody detecting tests were performed to check for an AE with the patient's serum (7). A tissue-based assay was initially performed to screen for AE. Then, cell-based immunoassay and immunoblotting were performed to detect synaptic and intracellular auto-antibodies. In cases of an unknown auto-antibody being identified in the tissue-based assay, further tests were performed by staining cultured neuronal cells with the patients' serum. In addition, immunoprecipitation and mass spectrometry were performed to identify novel specific antigens. Despite this extensive testing of AE, no auto-antibodies were identified.

We suspected antibody-negative AE and started first-line treatment with high-dose intravenous methylprednisolone (1,000 mg) for 5 days, followed by oral prednisolone (Figure 1G). However, the patient's status deteriorated; he presented with episodes of right facial grimacing and right upper limb contraction, consistent with faciobrachial dystonic seizures. Continuous electroencephalogram demonstrated rhythmic sharp-and-waves arising from the left temporo-occipital lobe. These alterations were controlled with levetiracetam, valproic acid, and lacosamide.

The patient further deteriorated to a comatose state (GCS: E1VtM1) with generalized tonic-clonic seizures, requiring intravenous immunoglobulin (IVIg, 1 mg/kg for 5 days) on hospital day 16. His arousal level plateaued (GCS: E1VtM1) after receiving IVIg for 2 weeks. We then administered a second-line treatment, rituximab (IV, 375 mg/m^2^, once weekly × 4 doses); before rituximab administration, we checked the patient's B-cell counts (CD19+/CD20+) on hospital day 28. CD19+/CD20+ counts of total lymphocytes in the peripheral blood were 15.3 and 15.1%, respectively (Figure 1G). After receiving rituximab for 3 weeks, the patient's mental status started improving (GCS: E4VtM5), and follow-up brain MRI findings were markedly improved (Figures 1C–F). The patient was discharged to a rehabilitation center (GCS: E4VtM6). At the last follow-up 5 months later, CD19+/CD20+ B-cells both remained at 0% (GCS: E4V5M6).

## Discussion

We reported a case of antibody-negative AE, with an association among clinical symptoms, CD19+/CD20+, and brain MRI findings, and treatment response. Moreover, we propose that rituximab could be considered a crucial treatment for antibody-negative AE when a patient exhibits unexplained symptoms. As antibody-negative AE is rare, we will establish a registry to collect data on its symptoms, treatments, and outcomes.

Clinical presentation-based diagnosis is more robust when considered with CSF, EEG, and MRI findings to guide antibody-negative AE treatment and ensure good response with immunotherapy (7). Owing to the overlapping clinical features, ruling out diseases that mimic AE in patients with severe neurological deficits is critical before initiating immunotherapy. Crucially, more than half of AEs are antibody-negative; nonetheless, some suspicion is required to reach the diagnosis of antibody-negative AE, and prompt management is needed to treat a potentially irreversible condition (7). The appropriate therapy should treat autoimmune phenomena against cell membranes, synapses, or intracellular antigens in the brain, similar to those with antibody-positive AE (1, 8).

Treatment options for antibody-negative AE, similar to antibody-positive AE, are broadly immune-suppressing agents for various specific steps in the pathogenesis of AE (9). As in other autoimmune disorders, corticosteroids are used in the treatment of antibody-negative AE to inhibit the inflammatory process. However, corticosteroids might have less specificity with several systemic side effects, and their efficacy is limited in AE (9). Other therapeutic approaches include various auto-antibodies and diverse immune mediators (e.g., IVIg, plasma exchange), B cells and short-lived plasma cells (e.g., rituximab), and specific cytokines (e.g., tocilizumab); anti-proliferative agents (e.g., cyclophosphamide, azathioprine, mycophenolate mofetil) are also used therapeutically (9).

B cells, in particular, present the main mechanism in autoimmune etiology (10), as demonstrated by the clinical success of B cell depletion therapies (BCDT). BCDTs such as the targeting CD20+, CD19+, and BAFF are used to treat autoimmune diseases. Although antibody-secretion is a negative consequence in autoimmune disease, BCDT impacts these cells and associated antibody levels. Therefore, in our case, it might be appropriate to evaluate B-cell counts to assess the treatment response to rituximab and formulate a prognosis. The improvements in consciousness level, MRI findings, and CD19+/CD20+ counts seemed related; however, they were independent of the antibody status.

This case report considered an AE etiology since the patient's differential diagnosis presenting with clinical symptoms, and MRI results suggested probable AE, even though the patient was antibody-negative. We speculate that rituximab played a crucial role in controlling the adverse autoimmune event before the end of the half-life of the first-line treatment, preventing the maturation of pre-B cells and B-cell depletion (7). Also, AE may require regular follow-up to evaluate symptoms or other diseases that may change the management strategy.

A limitation of our study is that antibody-negative AE, like other autoimmune diseases, has few biomarkers to identify the underlying mechanisms. Therefore, we cannot rule out the possibility of yet unknown antibodies that may be undetectable with the techniques used here. In addition, it can be difficult to distinguish between AEs because the patient's symptoms are similar to the other antibody-positive AEs such as LGI AE. Moreover, the improvement of the patient's symptoms may be attributed to the effects of IVIg rather than rituximab. Nevertheless, we proceeded with treatment by following the treatment guideline as much as possible. Therefore, our experience could be helpful to clinicians for real-life information on the use of rituximab for AE. In particular, our results and others could be useful for planning future comparative effectiveness studies with rituximab.

## Data Availability Statement

The original contributions presented in the study are included in the article/supplementary material, further inquiries can be directed to the corresponding author.

## Ethics Statement

The studies involving human participants were reviewed and approved by this case report was conducted according to the tenets of the Declaration of Helsinki and approved by the Institutional Review Board of Inha University Hospital (2021-01-025). Our patient's family provided informed written consent. Written informed consent was obtained from the individual for the publication of any potentially identifiable images or data included in this article.

## Author Contributions

S-HP and Y-CK: conception and organization of the research project, analysis and interpretation, and writing of the first draft of the manuscript. S-HP: execution of the research project and review and critique of the manuscript. All authors contributed to the article and approved the submitted version.

## Conflict of Interest

The authors declare that the research was conducted in the absence of any commercial or financial relationships that could be construed as a potential conflict of interest.

## Publisher's Note

All claims expressed in this article are solely those of the authors and do not necessarily represent those of their affiliated organizations, or those of the publisher, the editors and the reviewers. Any product that may be evaluated in this article, or claim that may be made by its manufacturer, is not guaranteed or endorsed by the publisher.
